# Geographic Variations in Retention in Care among HIV-Infected Adults in the United States

**DOI:** 10.1371/journal.pone.0146119

**Published:** 2016-01-11

**Authors:** Peter F. Rebeiro, Stephen J. Gange, Michael A. Horberg, Alison G. Abraham, Sonia Napravnik, Hasina Samji, Baligh R. Yehia, Keri N. Althoff, Richard D. Moore, Mari M. Kitahata, Timothy R. Sterling, Frank C. Curriero, for the North American AIDS Cohort Collaboration on Research and Design (NA-ACCORD)

**Affiliations:** 1 Vanderbilt University School of Medicine, Nashville, Tennessee, United States of America; 2 Johns Hopkins University, Baltimore, Maryland, United States of America; 3 Mid-Atlantic Permanente Research Institute, Kaiser Permanente Mid-Atlantic States, Rockville, Maryland, United States of America; 4 University of North Carolina at Chapel Hill, Chapel Hill, North Carolina, United States of America; 5 British Columbia Centre for Excellence in HIV/AIDS, Vancouver, British Columbia, Canada; 6 University of Pennsylvania, Perelman School of Medicine, Philadelphia, Pennsylvania, United States of America; 7 University of Washington School of Medicine, Seattle, Washington, United States of America; Boston University, UNITED STATES

## Abstract

**Objective:**

To understand geographic variations in clinical retention, a central component of the HIV care continuum and key to improving individual- and population-level HIV outcomes.

**Design:**

We evaluated retention by US region in a retrospective observational study.

**Methods:**

Adults receiving care from 2000–2010 in 12 clinical cohorts of the North American AIDS Cohort Collaboration on Research and Design (NA-ACCORD) contributed data. Individuals were assigned to Centers for Disease Control and Prevention (CDC)-defined regions by residential data (10 cohorts) and clinic location as proxy (2 cohorts). Retention was ≥2 primary HIV outpatient visits within a calendar year, >90 days apart. Trends and regional differences were analyzed using modified Poisson regression with clustering, adjusting for time in care, age, sex, race/ethnicity, and HIV risk, and stratified by baseline CD4+ count.

**Results:**

Among 78,993 adults with 444,212 person-years of follow-up, median time in care was 7 years (Interquartile Range: 4–9). Retention increased from 2000 to 2010: from 73% (5,000/6,875) to 85% (7,189/8,462) in the Northeast, 75% (1,778/2,356) to 87% (1,630/1,880) in the Midwest, 68% (8,451/12,417) to 80% (9,892/12,304) in the South, and 68% (5,147/7,520) to 72% (6,401/8,895) in the West. In adjusted analyses, retention improved over time in all regions (p<0.01, trend), although the average percent retained lagged in the West and South vs. the Northeast (p<0.01).

**Conclusions:**

In our population, retention improved, though regional differences persisted even after adjusting for demographic and HIV risk factors. These data demonstrate regional differences in the US which may affect patient care, despite national care recommendations.

## Introduction

Mapping health outcomes and identifying geographic variations in care are useful tools in public health, assisting decision-makers in identifying locales in greatest need of resources.[1–7] The field of HIV epidemiology has been no exception, and analyses of geographic variation in HIV incidence, extent, severity, and intervention effectiveness have yielded insights into the changing nature and trajectory of the pandemic.[8–16] In the U.S., HIV prevalence, incidence, disease progression, treatment, and mortality have been noted to differ across geographic regions and individual states.[17–21]

Retention in care is associated with improved access to antiretroviral therapy (ART), greater likelihood of virologic suppression, and less rapid HIV disease progression.[22–27] Similarly, the same demographic, clinical, and socioeconomic factors (i.e., younger age, Black race, higher CD4 count, and unstable housing status) have been repeatedly associated with suboptimal retention in various contexts. However, these analyses have rarely focused on geographic heterogeneity as a potential source of clinical retention differences and have incorporated these data by adjusting for clinic site in multi-site analyses or examining relatively small numbers of jurisdictions.[28–39] Further, some of the studies in which these patterns of retention were discerned may have cohort-specific traits which could affect clinic attendance such as state Medicaid funding levels or local social stigmas (e.g., a history of intolerance toward sexual minorities) that could limit their external generalizability to persons living with HIV/AIDS (PLWHA) in the U.S. Nevertheless, recent major policy initiatives, including the National HIV/AIDS Strategy (NHAS), have identified improving clinical retention, and targeting impediments to these improvements, as goals central to improving outcomes across the HIV Care Continuum in the U.S.[40–43]

Yet retention in care requires consistent and ongoing interaction with the healthcare system, a process which may include various obstacles which differ geographically (due to demographic, economic, risk behavior, political, and cultural factors).[9,20,21,35,44] In consideration of these issues, our aim was to describe the geography of clinical care experience over time in order to provide evidence for evaluating benchmarks of national HIV policy goals and to better understand factors that are pertinent to public health interventions designed to improve retention in HIV care.[42] We therefore quantified the geographic heterogeneity of retention between 2000 and 2010 within a large and geographically diverse HIV cohort that is demographically similar to PLWHA in the U.S.[45]

## Methods

### Population and study design

The North American AIDS Cohort Collaboration on Research and Design (NA-ACCORD) represents the United States and Canada in the International epidemiologic Databases to Evaluate AIDS (IeDEA) project. The NA-ACCORD began collecting data from multi- and single-site interval and clinical cohorts in 2006.[46] The Institute of Medicine of the National Academies (IOM) has promulgated the NA-ACCORD, due to its size and demographic similarity with PLWHA in the U.S., as one of 12 data systems appropriate to assess quality of care goals, such as improving clinical retention, in the NHAS and Affordable Care Act [24] Details of the data collection and submission process for the NA-ACCORD have been published previously.[47] Briefly, clinical, demographic, and geographic data from 25 cohorts are transmitted to a centrally-administered Data Management Core annually where all contributed data are harmonized. Data undergo quality control for completeness and accuracy, including measures to reduce the probability that an individual was concurrently participating in more than one clinical cohort. The activities of the NA-ACCORD and each of its participating cohorts have been reviewed and approved by their respective local institutional review boards and by the Johns Hopkins School of Medicine institutional review board. Written consent was either obtained individually, or else waivers of individual consent were obtained through each site’s respective local institutional review board. All data were de-identified prior to analysis.

Among clinical cohorts, only patients with ≥ 2 HIV primary care visits within 12 months were enrolled into the NA-ACCORD, limiting the NA-ACCORD population to patients successfully linked to and established as “in care” proximal to cohort entry.[24,26]

Adult participants who had ≥ 1 HIV primary care visit between January 2000 and December 2010 were included in this longitudinal, retrospective cohort study. Classic prospective cohorts were excluded to allow an exclusive focus on patterns of patient clinical care in clinical cohorts. Canadian cohorts were also excluded. The 12 included clinical cohorts were comprised of patients from all 50 U.S. states, Washington, D.C., and Puerto Rico, 818 of 887 US 3-digit ZIP Code Tabulation Areas (ZCTA), and 167 clinical sites located in areas of dense population across the country.

Our nested study was reviewed by the Johns Hopkins Bloomberg School of Public Health institutional review board, and it was deemed non-human-subject/non-research because it was a secondary analysis of existing, anonymized/de-identified data.

### Retention measures, factors associated with retention, and follow-up

The outcome for our analysis was clinical retention in care (“retention”), defined using the IOM indicator: ≥ 2 HIV primary care visits within a calendar year, ≥ 90 days apart.[24] This measure was used because it is the same as that adopted in the National HIV/AIDS Strategy. We have previously evaluated the strong agreement of this indicator with DHHS indicators and with laboratory proxies.[25,48] Only HIV primary care encounters were used to define retention. Other outpatient encounter types, including subspecialty care, emergency department, dental, mental health, nursing, nutrition, orientation, pharmacy, social work/case manager, substance abuse, and other/unknown outpatient encounters, were not used for these analyses. Inpatient visits and laboratory only visits were also excluded.

Participant age (<40, 40–49, 50–59, and ≥60 years), sex, race/ethnicity (White, Black, Hispanic, or other/unknown), HIV acquisition risk factor (male sexual contact with men (MSM), injection drug use [IDU], heterosexual contact, or other/unknown), receipt of ART for ≥6 months in a year (≥3 antiretroviral drugs from ≥2 classes, or a triple nucleoside/nucleotide reverse transcriptase inhibitor (NRTI) regimen containing abacavir or tenofovir), first CD4+ cell count during each year of follow-up, last HIV-1 RNA measure during each year of follow-up, and geographic location of residence (US Centers for Disease Control (CDC)-defined region, state, and 3-digit ZCTA) were included in analyses of factors by which clinical retention may have differed. The composition of regions by state is described below.

ART receipt and CD4+ cell count were used in secondary analyses as baseline covariates for stratification indicating health status and access to care at cohort enrollment; HIV-1 RNA was excluded from regression analyses due to its putative role as time-dependent confounder in the longitudinal relationship between demographic and HIV risk characteristics and retention. Geographic location of patient residence was collected at cohort entry and was not allowed to vary over time due to limited availability of residential movement data over the study period.

Individual data were summarized into one observation per year between 2000 (at the earliest) and the final encounter prior to the end of 2010. The initial year of care was excluded if the patient entered in the final quarter of a calendar year (and were thus ineligible to be “retained” in their year of entry). Year of death during the study period was excluded from analyses due to individuals not being uniformly “at risk” for successful retention in that year. Follow-up time ranged between 1 and 11 person-years, and individuals contributed multiple outcomes while under observation.

### Additional geographic information

Location of patient residence by ZCTA was collected by individual clinical cohorts. Data consistency checks were performed to ensure that ZCTA of residence corresponded correctly with state of residence (as ZCTAs may be aggregated to the state-level along coterminous boundaries). State of residence was used to assign patients to geographic regions of the U.S. based on CDC and US Census Bureau definitions. US CDC-defined regions were: Northeast: CT, ME, MA, NH, NJ, NY, PA, RI, VT; Midwest: IL, IN, IA, KS, MI, MN, MO, NE, ND, OH, SD, WI; South: AL, AR, DE, DC, FL, GA, KY, LA, MD, MS, NC, OK, SC, TN, TX, VA, WV; and West: AK, AZ, CA, CO, HI, ID, MT, NV, NM, OR, UT, WA, WY.[49] Demographic, geographic, and economic characteristics of ZCTAs were derived from 2000 and 2010 US decennial census estimates and assigned as follows: for years 2000–2003: from the 2000 census; for years 2008–2010: from the 2010 census; and for years 2004–2007: mid-point estimates between census years 2000 and 2010. Census-derived population-level variables included median age within the ZCTA and proportions of the ZCTA that were of female sex, of Black race, residing in a rural area, and living below the Federal poverty level. Rural areas and the poverty level were defined according to US Census Bureau standards.[50] For ZCTA-level analyses, individual characteristics from NA-ACCORD participants were aggregated to the ZCTA level as median age in the sample, and the proportion of the sample that were of female sex, of Black race, and that had IDU as an HIV risk factor within the ZCTA.

For participants from 2 clinical cohorts (N = 35,131) whose residential data was unavailable, clinic location was used as a proxy for state of residence, but not for ZCTA; individuals from these cohorts were included in descriptions and analyses of regional and state-level differences in retention but not in analyses using ZCTA-level data.

### Statistical models and methods

Regional differences in the percentage of patients clinically retained within strata of demographic and clinical characteristics were detected by χ^2^ test. Modified Poisson regression using a Generalized Estimating Equation (GEE) was used to assess temporal trends and determine the relative risks (RR) and 95% confidence intervals (CI) of retention based on demographic and geographic factors at the individual level.[51] An unstructured working correlation was used for repeated outcomes within individuals in the GEE regression.[52,53] Time during the study period (i.e., study year) was included in models as a restricted cubic spline with 3 knots (at 2, 6, and 10 years) and as a categorical term for predictive margins.[54] All individual-level models were also adjusted for total time contribution to the study (to account for possible cohort effects) and for contributing cohort site (to account for potential clinical practice differences). All proportions at the ZCTA level were mean-centered to ease interpretation in logistic regression modeling using GEE. In secondary analyses, the relationship between baseline CD4+ count and ART use with retention were assessed to explore regional differences in retention trajectories by initial access to care and late diagnosis. Interaction terms for region-by-baseline factors were included with the full Poisson regression model used in primary analyses at the individual level. Missing baseline CD4+ counts were multiply imputed using a predictive mean model regression including baseline age, sex, race/ethnicity, HIV risk factor, ART use, and cohort site.[55]

Population-averaged effects are generally viewed as more germane to policy decisions, and there is evidence that mixed effects approaches may induce bias in this context.[56,57] Thus, the population-averaged model was employed here. ZCTA-level differences in proportions retained were also explored using logistic regression with GEE, adjusting for aggregated individual and ZCTA-level census characteristics.[58,59] Additional details of Poisson and logistic regression models with GEE at the individual and ZCTA levels, respectively, are available in the Appendix (S1 File). Maps were generated using ArcGIS version 10.1 (Environmental Systems Research Institute, Redlands, CA) and statistical analyses were performed using Stata version 12.1 (StataCorp, College Station, TX).

## Results

Among 78,993 adults with 444,212 person-years of follow-up, the median time in care was 7 (Interquartile Range: 4–9) years. The Midwest sample had the smallest percentage of regional prevalent HIV cases with 3.7% (3,583/97,711); the West had the highest percentage with 7.5% (12,037/159,523), using CDC data for prevalent HIV cases from 2009 as the denominator (the last year in which all 12 clinical cohorts contributed data).[49] There were significant differences between US geographic regions in the percentage of person-time retained for each demographic and clinical characteristic evaluated (Table 1).

**Table 1:** Percent of person-years successfully retained (with % person-years contributed) in the NA-ACCORD, defined by encounters, stratified by demographic, clinical, and geographic characteristics, from 2000 through 2010.

	Total	Northeast	Midwest	South	West
	Person-Years (PY)	% PY Retained	% PY Retained	% PY Retained	% PY Retained
Factor	(% PY ctrbtd.)	(% PY ctrbtd.)	(% PY ctrbtd.)	(% PY ctrbtd.)	(% PY ctrbtd.)
Total	7 444,212 (100)	78 (22)	81 (7)	73 (45)	75 (26)
Age (years)*					
≤39	118,626 (27)	69 (24)	72 (24)	63 (28)	62 (27)
40–49	166,389 (37)	77 (38)	81 (37)	73 (36)	70 (39)
50–59	114,784 (26)	83 (28)	84 (27)	80 (26)	76 (24)
≥60	44,413 (10)	88 (10)	89 (12)	88 (10)	83 (10)
Sex					
Male	367,048 (83)	78 (74)	80 (86)	74 (82)	71 (90)
Female	77,164 (17)	77 (26)	83 (14)	69 (18)	67 (10)
Race/Ethnicity					
Non-Hisp. White	183,580 (41)	80 (28)	83 (45)	77 (36)	71 (62)
Non-Hisp. Black	194,787 (44)	77 (51)	78 (46)	71 (55)	68 (18)
Hispanic	48,125 (11)	78 (19)	84 (4)	73 (7)	71 (12)
Other/Unk.	17,720 (4)	70 (2)	81 (5)	72 (2)	67 (8)
HIV Risk Factor					
MSM	152,691 (34)	79 (28)	83 (30)	73 (28)	69 (52)
IDU	86,301 (19)	76 (26)	76 (18)	72 (20)	69 (14)
Hetero	95,869 (22)	78 (30)	82 (17)	68 (23)	66 (13)
Other/Unk.	109,351 (25)	78 (16)	81 (35)	79 (29)	77 (21)
CD4+ Cell Count (cells/mm^3^)^a^					
<200	73,559 (17)	81 (16)	85 (15)	74 (18)	76 (15)
200–349	75,340 (17)	84 (18)	87 (15)	79 (17)	74 (17)
350–499	77,588 (17)	85 (17)	89 (16)	81 (17)	74 (19)
≥500	134,202 (30)	86 (30)	90 (31)	82 (28)	74 (33)
Missing	83,523 (19)	49 (19)	56 (23)	50 (20)	47 (16)
HIV-1 RNA (copies/mL)^b^					
≥200 copies	178,803 (40)	76 (41)	81 (38)	70 (44)	68 (34)
<200 copies	198,980 (45)	88 (47)	92 (42)	86 (39)	77 (54)
Missing	66,429 (15)	46 (12)	56 (20)	54 (17)	46 (13)
ART Receipt (≥6 months/year)					
<6 months ART	170,251 (38)	59 (37)	62 (37)	55 (41)	53 (35)
≥6 months ART	273,961 (62)	88 (63)	92 (63)	86 (59)	80 (65)

Percent of person-years retained during the study by encounter (i.e., years “in care” between cohort entry and final encounter) is different by region within every stratum (χ^2^ test, p<0.01)

*: age at cohort enrollment

^a^: at the first measurement in each calendar year during follow-up;

^b^: at the last measurement in each calendar year during follow-up

Region information missing for residents of Puerto Rico (N = 255), the US Virgin Islands (5), or where state-level residence was missing (N = 12)

Ctrbtd.: contributed; MSM: male sexual contact with men; IDU: injection drug use; Hetero: heterosexual contact; ART: antiretroviral therapy (≥3 agents from ≥2 classes or a triple-NRTI regimen containing abacavir or tenofovir)

**Fig 1:**
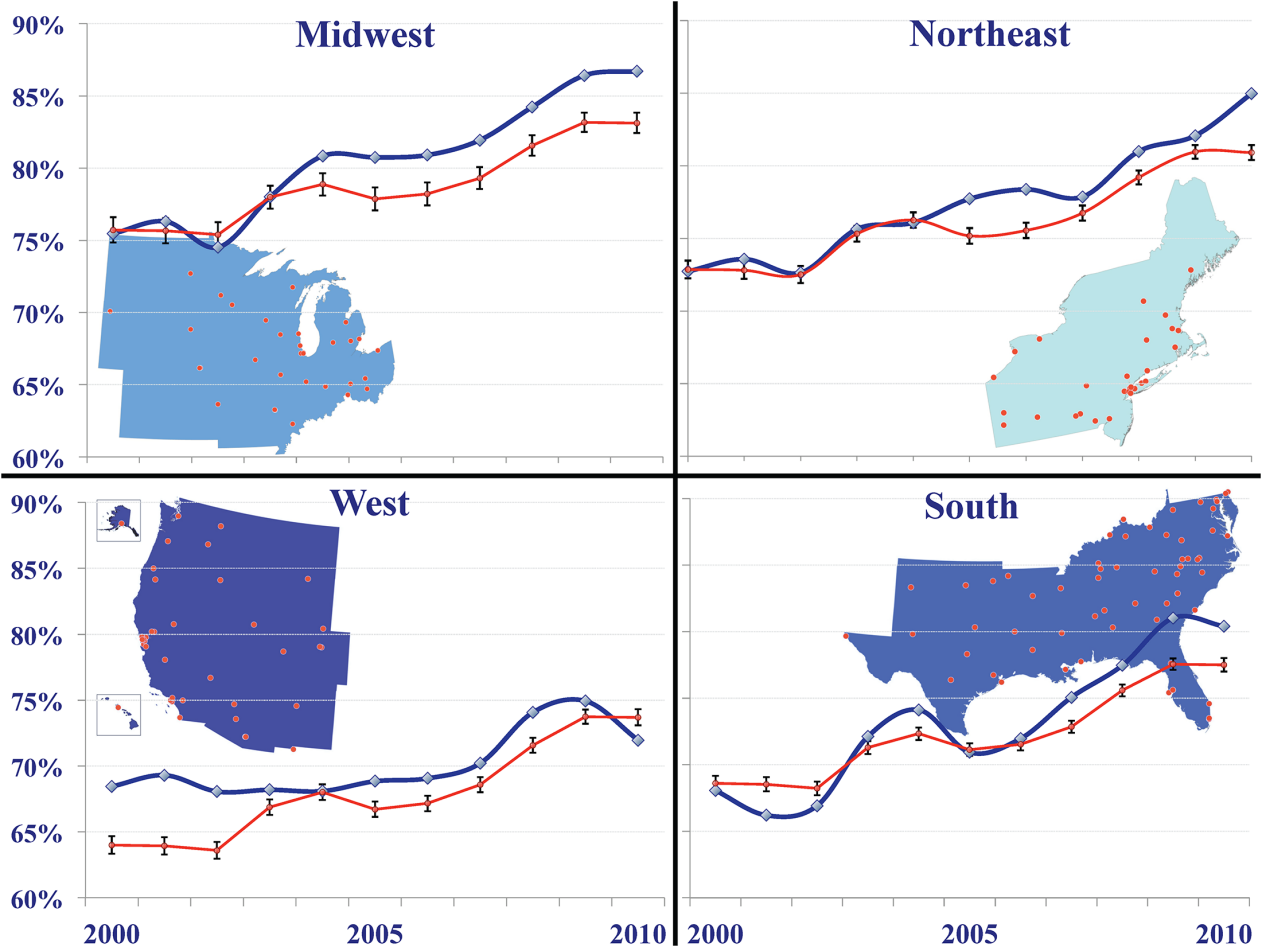
Temporal trends in percentage of individuals successfully clinically retained in the NA-ACCORD by CDC-defined region of the United States, from 2000–2010, by CDC-defined region of the United States. Diamonds are National HIV/AIDS Strategy/Institute of Medicine retention indicator percentages (≥2 visits in a calendar year, >90 days apart). Circles are Predictive Margins for the Probability of Being Retained by IOM indicator using a Region-by-Time interaction effect (Fully Adjusted Logistic Model with GEE) U.S. Centers for Disease Control and Prevention (CDC)-defined Regions: *Northeast*: CT, ME, MA, NH, NJ, NY, PA, RI, VT; *Midwest*: IL, IN, IA, KS, MI, MN, MO, NE, ND, OH, SD, WI; *South*: AL, AR, DE, DC, FL, GA, KY, LA, MD, MS, NC, OK, SC, TN, TX, VA, WV; *West*: AK, AZ, CA, CO, HI, ID, MT, NV, NM, OR, UT, WA, WY.

The percentage of individuals successfully retained increased from 2000 to 2010 in each region: from 73% (5,000/6,875) to 85% (7,189/8,462) in the Northeast, 75% (1,778/2,356) to 87% (1,630/1,880) in the Midwest, 68% (8,451/12,417) to 80% (9,892/12,304) in the South, and 68% (5,147/7,520) to 72% (6,401/8,895) in the West (Fig 1).

**Fig 2:**
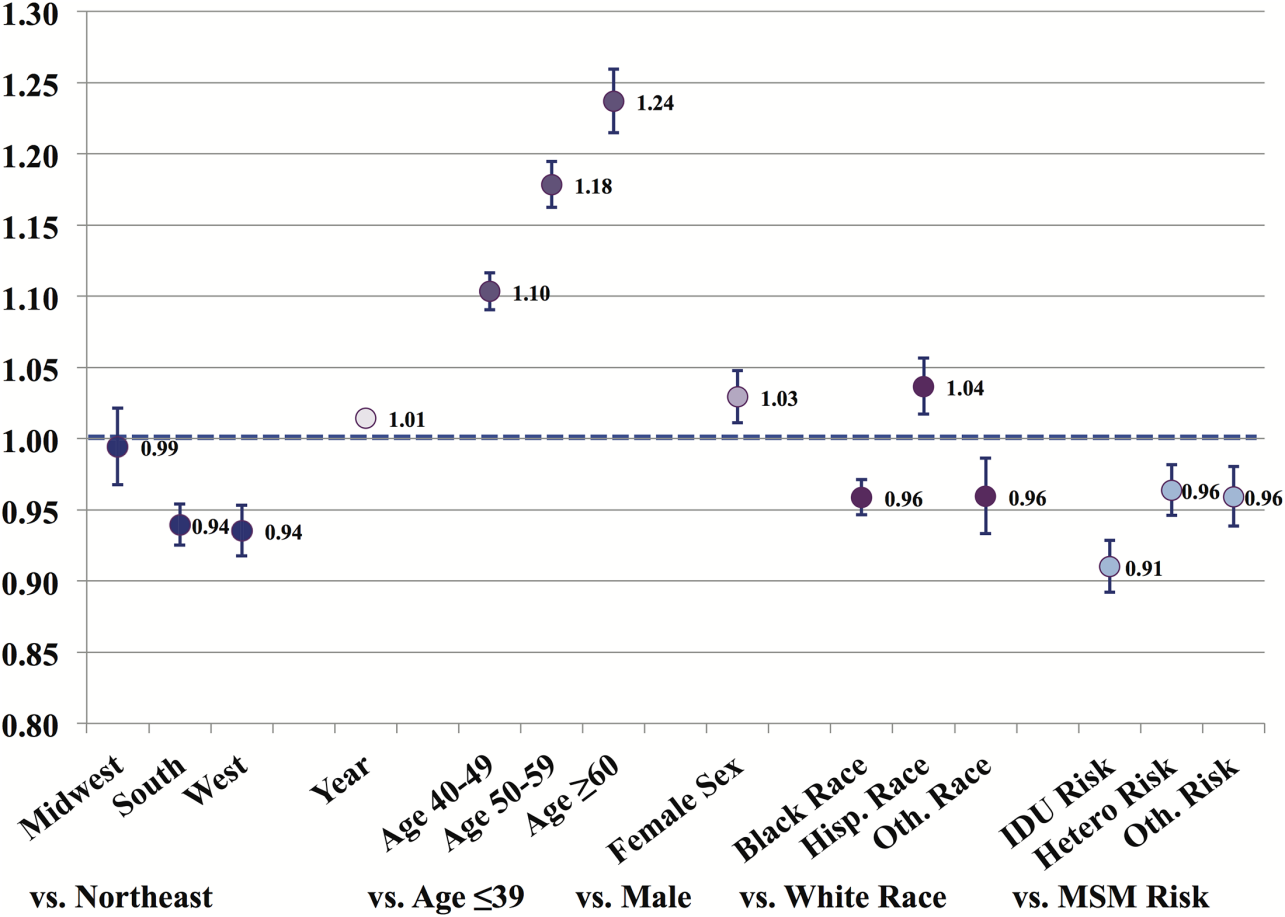
Risk Ratio estimates and 95% Confidence Intervals for factors associated with retention. Results from modified Poisson regression model using a Generalized Estimating Equation and adjusting for total time in care.

In adjusted regression models, there was a trend for improved retention over time (p<0.01) for all regions. Over the study period, the probability of retention was lower in the West (Risk Ratio (RR): 0.94; 95% Confidence Interval (CI): 0.92–0.95) and South (RR: 0.94; 95% CI: 0.93–0.95), and no different in the Midwest (RR: 1.02; 95% CI: 1.00–1.05), compared to the Northeast (p<0.01). Other factors including younger age, male sex, Black race, and IDU and Heterosexual contact as HIV acquisition risk factors were significantly associated with a decreased likelihood of retention (Fig 2).

We found significant effect modification for racial differences in retention by region, in which the risk of retention among Black patients was poorer relative to White patients in the South than in other regions (RR: 0.94; 95% CI: 0.93–0.96, for Black vs. White patients in the South vs. the Northeast). We also found significant associations of baseline CD4+ count with retention within the Northeast, South, and West. However, the association in the Northeast (RR: 1.03 for both 200–349 and for ≥500 vs. <200 cells/μL; p = 0.02 each) was in the opposite direction of the relationship in the South and West (higher baseline CD4+ count was associated with between 3 and 6% decreased probability of retention vs. <200 cells/μL; p≤0.01 each, Table A in S1 File). Further, receipt of ART at baseline was associated with between 22 and 30% increased probability of retention within each region (p<0.01 each, Table A in S1 File). In each of these secondary analyses, the magnitude, direction, and significance of the associations between age group, sex, race, HIV risk factor, geographic region and retention were similar to those in the primary analysis (data not shown). Though state-level patterns generally conformed to the regional patterns observed (Southern and Western states lagged in observed and predicted retention probabilities over time), adjusting for state in the full model did not improve the model fit considerably. (Part F of Fig A in S1 File).

In models at the ZCTA-level, only an increase in median age (Odds Ratio (OR): 1.10 per year; 95% CI: 1.09–1.11) and a decrease in the census-based proportion of a ZCTA that were of Black race (OR: 0.36 per percentage difference increase from the mean proportion; 95% CI: 0.15–0.87) were significantly associated with improved retention (Table 2).

**Table 2:** Estimated _95%_ Odds Ratios _CIs_ for retention from ZCTA-level regression models.

	Full Model			Model 2			Model 3			Model 4		
QIC (Adjusted)	8724.7			8724.7			8722.8			8722.3		
Median Age (Yrs.)	_1.09_	**1.10**	_1.11_	_1.09_	**1.10**	_1.11_	_1.09_	**1.10**	_1.11_	_1.09_	**1.10**	_1.11_
Sample Proportion Female Sex	_0.74_	_2.39_	_7.72_	_0.75_	_2.40_	_7.72_	_0.75_	_2.39_	_7.72_			
Sample Proportion Black Race	_0.39_	_0.65_	_1.09_	_0.39_	_0.65_	_1.09_	_0.39_	_0.65_	_1.09_	_0.40_	_0.65_	_1.09_
Sample Proportion IDU Risk	_0.64_	_1.03_	_1.68_	_0.64_	_1.03_	_1.68_	_0.64_	_1.03_	_1.68_	_0.67_	_1.08_	_1.75_
Census Proportion Female Sex	_0.00_	_10.80_	_48302.1_									
Census Proportion Black Race	_0.15_	**0.36**	_0.87_	_0.16_	**0.38**	0.90	0.17	**0.37**	0.82	_0.17_	**0.37**	_0.83_
Census Proportion Rural Area	_0.67_	_1.01_	_1.53_	_0.66_	0.97	1.46	0.66	0.97	1.43	_0.67_	0.98	_1.44_
Census Proportion Living Below Poverty Line	_0.14_	_0.87_	_5.38_	_0.16_	0.94	5.70						
Region												
Northeast	Reference			Reference			Reference			Reference		
Midwest	_0.75_	_0.96_	_1.26_	_0.74_	_0.95_	_1.24_	_0.74_	_0.95_	_1.24_	_0.74_	_0.95_	_1.24_
South	_0.88_	_1.14_	_1.48_	_0.87_	_1.12_	_1.46_	_0.87_	_1.12_	_1.45_	_0.87_	_1.12_	_1.45_
West	_0.68_	_0.92_	_1.25_	_0.68_	_0.89_	_1.18_	_0.68_	_0.89_	_1.17_	_0.68_	_0.89_	_1.17_

**Bold** point estimates are statistically significant (p<0.05)

QIC: the Quasi-likelihood Information Criterion of Pan (a measure of model fit, compared with an independent working correlation structure)

All logistic regression models used GEE with an unstructured working correlation structure, and all sample and census proportions were mean-centered

Increasing total time in care was also significantly associated with improved retention (OR: 1.00, 95% CI: 1.00–1.01, for all models)

## Discussion

We found that among PLWHA successfully linked to and enrolled in care, the percentage of patients who were retained in care improved over time for all regions, with 72–87% retained in care in 2010. Despite this finding, the South and West exhibited lower retention levels over time compared to the Northeast. The results regarding the South are of particular concern in that they are troublingly consistent with surveillance and prior studies showing worse HIV outcomes, and significant sex and race disparities in these outcomes, compared to the rest of the U.S.[17,19–22] In the South, with its history of poorer educational performance, poorer access to healthcare than much of the rest of the country, and regressive policies with respect to racial/ethnic and sexual minorities, the lag in improved quality of care outcomes could be the product of several forces.[17,18,21] A geographically dispersed epidemic with residents of more rural areas who have longer commutes to clinic locations in primarily urban centers could affect the observed outcomes. Social stressors and structural factors that may limit the ability of individuals to access care (e.g., poverty, housing instability, discrimination, etc.) may also play an outsize role in the observed disparity of care between the South and Northeast, as noted in prior work.[21] However, further research will be required to explore the role of these factors in clinical care in this patient population.

Despite regional disparities, however, the improvement in retention adds further evidence to earlier research that showed increasing percentages of large linked and clinically-engaged populations being retained over long periods of follow-up in the U.S.[39,45,48] Notably, this upward trend persisted, even after adjusting for demographic, geographic, and HIV risk factor differences, and after accounting for differential contributions of time at risk for leaving care. This may have been due to increased attention to clinical retention during the study period or to increasing numbers of individuals placed on ART,[18,45] requiring increased engagement between patient and providers. Examining retention patterns by CD4+ count at cohort entry, a marker of delayed diagnosis or linkage to care, the South and West again exhibited decreased retention compared to the Northeast, though by similar magnitudes regardless of CD4+ stratum. This indicates that CD4+ count at entry to care did not significantly influence differences in retention over time between patients in the Northeast, South, and West, even though baseline CD4+ count differed between patients subsequently retained and not retained across the US.[38,39] Though this appears to bode well for efforts to improve retention among all presenters for HIV care, it remains a powerful indication that even among patients linked to care early in infection, there may remain structural factors that are region-specific which may dominate patients’ subsequent experiences of care.

Regarding ART use at cohort entry, an indicator of access to care, a fairly strong positive association between ART use and retention regardless of region may indicate the power of personal experience in engaging with healthcare services. This may include improved self-efficacy among patients and motivation to maintain contact with care providers once personally invested in their own medical care through ART use. However, it may also be the case that clinician decisions on initiating or continuing therapy favor patients more likely to improve their retention in care or those who have demonstrated stability in their clinic visit patterns (e.g., delaying ART initiation in IDU patients if they are actively using).[60] This does contradict reports of widespread clinician willingness to initiate therapy early in care among high risk patients, though in the largest such survey of clinician attitudes, the surveys were conducted only in New York and Washington, D.C.[61]

Many of the same groups that have been previously identified with suboptimal retention in care and inferior HIV outcomes in general, again emerged in this analysis. These groups include: younger individuals, Black patients, and those with IDU as HIV risk factor.[29,32,62–64] Even though the unadjusted retention rates among these groups approaches the NHAS goal of 80% (among Ryan White clients), there is room for improvement when compared to other HIV-infected sub-populations. Given those deficits, and the fact that risk networks, culture, and socioeconomics may differ across regions, states, and more granular geographic levels, the identification of locales that may benefit most from interventions to improve healthcare quality, access, and retention remains as important as ever. Clinicians and public health practitioners should note that though vulnerable and high burden groups identified again here may be obvious targets for interventions to improve access to and retention in care, their deficits may differ by region, and a “one size fits all” approach at the national (and perhaps even the regional) level may not be warranted. It may even be the case that simply expanding the use of ART at entry to care may improve patient engagement and retention. Further analysis will be required to isolate the potential effects of individual clinical decision-making before concluding that the observed relationship between ART use and retention is not due to confounding, though.

In this analysis, the regions analyzed were quite large, and economic, social, cultural, and political factors may vary widely even within regions (e.g., the most and least populous US states, California and Wyoming, respectively, are in the same geographic region- the West). Because within-region and between-region differences may play significant roles influencing differences in retention and other HIV outcomes, the monitoring of geographic trends in HIV clinical care, including all stages of the continuum, is an important activity to provide evidence for targeted outreach beyond a simple risk-group basis.

The additional analysis of ZCTA-level data, though providing a different lens through which to view demographic and HIV risk differences in retention in the US, add value in the additional layers of evidence they may provide to policy makers, particularly if individual-level data is unavailable.[65–67] Population-level differences in contextual factors such as poverty, crime, and housing instability may also be a desirable focus for policy when considering aggregate characteristics separately from individual characteristics related to care at a more granular level than the state or region. In this case, the differences observed are unlikely to stem from confounding by measured factors since the ZCTA-level analyses were adjusted using demographic and HIV risk characteristics, though they may be confounded by characteristics of the area of aggregation that were unavailable (such as public insurance income thresholds). Despite the limits in this context, these ZCTA-level analyses offer valuable insight into population-level processes such as persistent disparities in retention associated with regional age structure and racial composition that may be occurring in the care continuum at divisions smaller than the state level.

**Fig 3:**
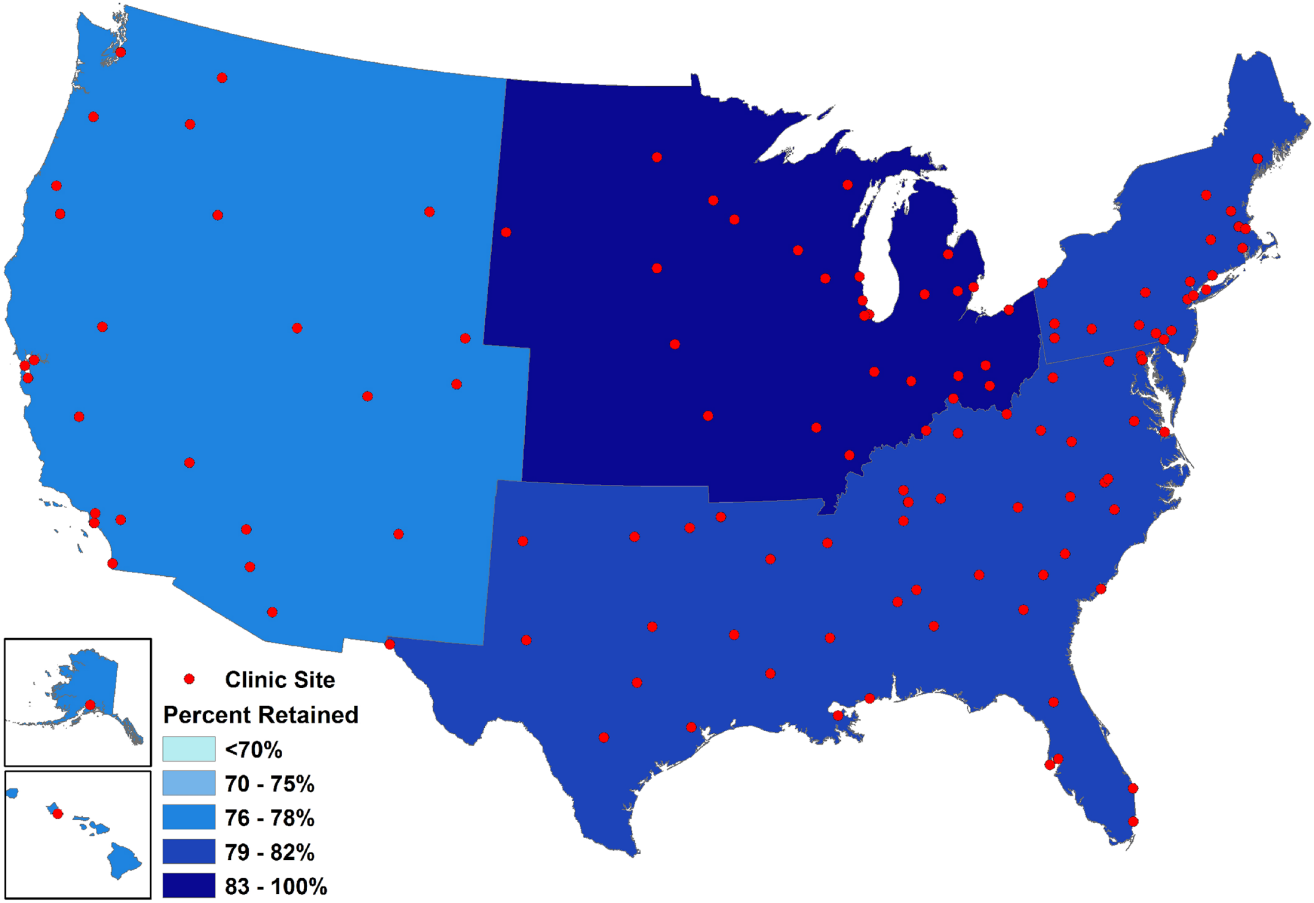
Region-level map of observed clinical retention status within the study sample in 2009 (N = 47,247), the final year in which all 12 clinical cohorts contributed data.

**Fig 4:**
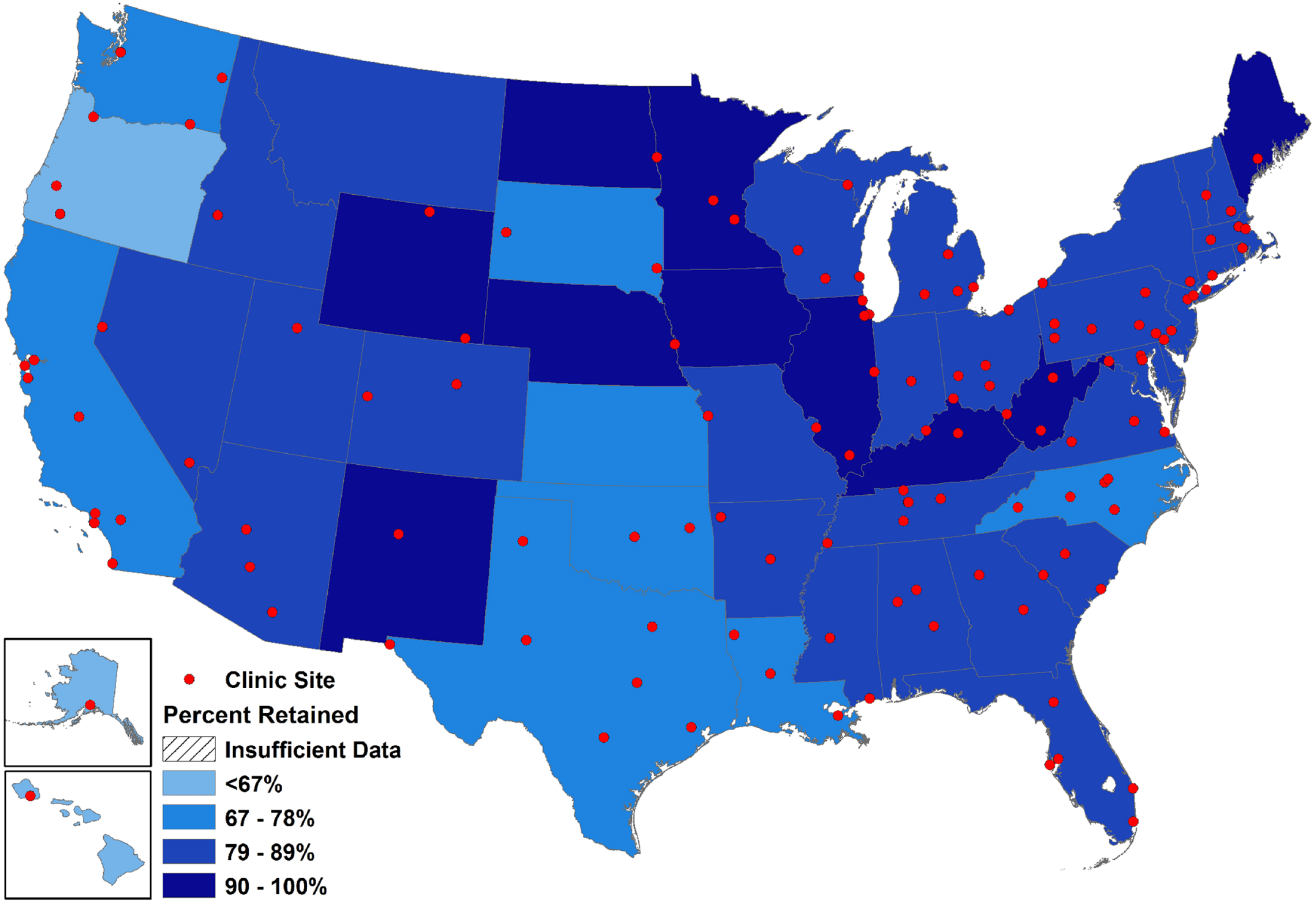
State-level map of observed clinical retention status within the study sample in 2009 (N = 47,247), the final year in which all 12 clinical cohorts contributed data.

**Fig 5:**
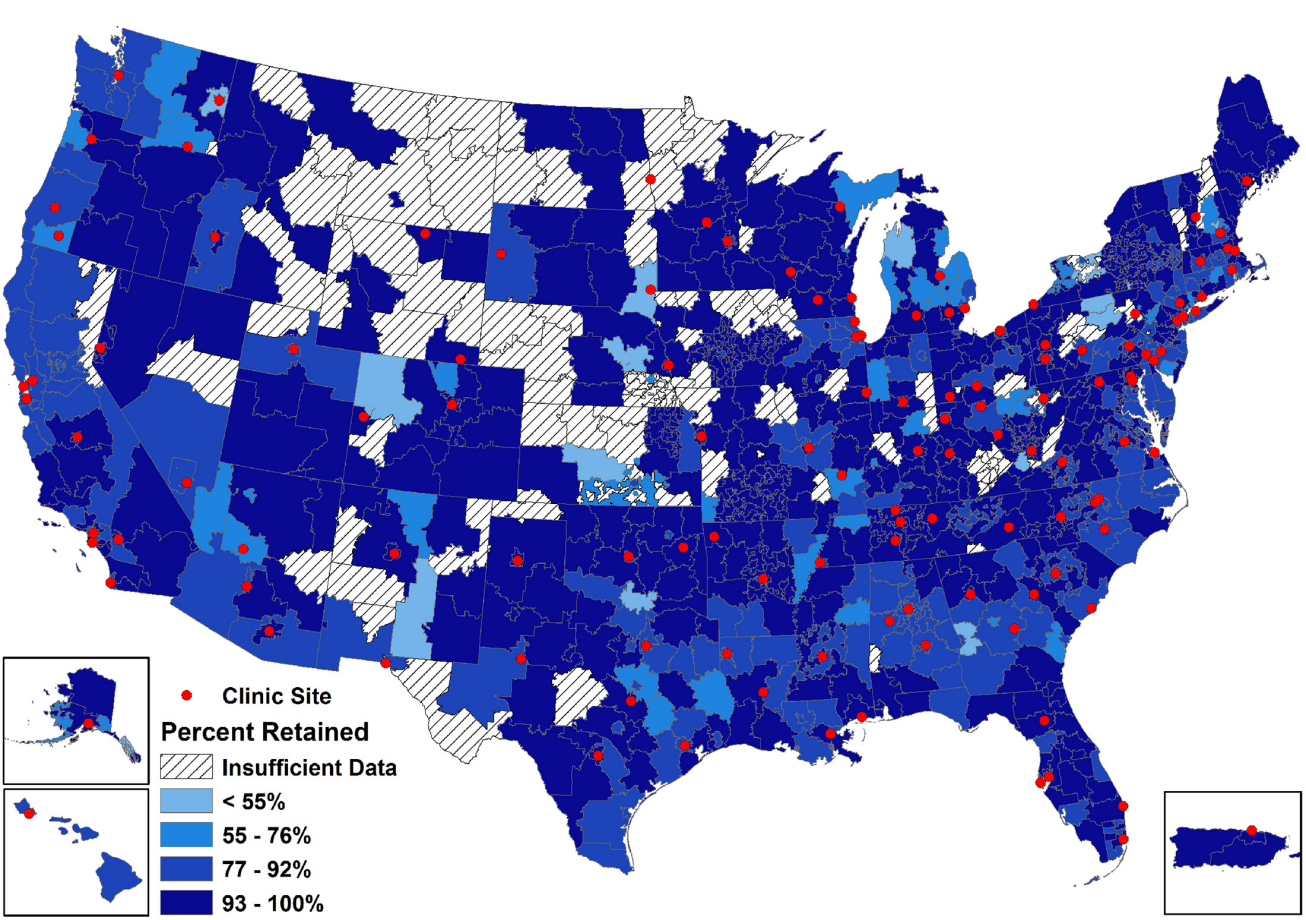
ZCTA-level map of observed clinical retention status within the study sample in 2009 (N = 47,247), the final year in which all 12 clinical cohorts contributed data.

Scrutinizing outcomes at varying levels of geographic resolution is useful (and perhaps necessary) for detecting patterns that may otherwise be obscured.[68] Noting retention patterns across ZCTA, state, and region levels, our work found fairly consistent trends, though the quality of inferences may not necessarily reflect the level at which public health actions are likely to be taken or at which effects may be most powerfully felt (Figs 3, 4 and 5). In the region-level map, the inter-regional disparities in retention are clearer, while increasing levels of magnification present a more complicated picture with the patterns less discernable. However, when describing disparate HIV quality of care outcomes, it may be particularly powerful to depict trends across areas that have been noted to have somewhat distinct cultures, political structures, and epidemic profiles, as denoted by the disaggregation of data across these very same regions in national HIV surveillance reports.[49] The assessment and mapping of these outcomes at various levels, which is itself unusual, provides a compelling depiction of these HIV outcomes in a more easily accessible and readable format for both scientific and lay audiences.

Further analysis incorporating smaller geographic divisions with richer individual- and population-level data (e.g., Medicaid status at the individual-level) will be required to better understand how geographic factors, and the social-structural characteristics they may be proxies of, impact retention in clinical care and HIV outcomes.

There were limitations in this analysis. First, medical insurance status and economic security are important factors influencing access to and retention in clinical care and which we did not have access to. We did, however, use census-derived proxies for socioeconomic data such as the proportion of the ZCTA that was rural (indicating longer travel to receive care) and the proportion of the ZCTA living below the Federal poverty line (indicating economic and insurance resources available to patients). Second, the group under observation was successfully linked to care and engaged at cohort enrollment, and therefore may not represent populations of greatest concern in the early stages of the continuum of care.

Using data from a large North American collaborative HIV cohort, our analysis demonstrated a persistent upward trend in retention in care across the U.S., but one that was differential in its extent by region. These differences correspond with observations of regional differences in other HIV outcomes and highlight the utility of geographic data and analyses in monitoring progress in the continuum of care on both a national and a local basis. Though policy prescriptions and public health actions such as expanded testing and prevention services or improved linkage and treatment programs may help stem the tide of the epidemic, knowing that they may be more urgently needed in the South or the West, as opposed to the Northeast, is surely a critical piece of information.[69]

## Supporting Information

S1 File (PDF)
