# Supplementary material for: Geographic Variations in Retention in Care among HIV-Infected Adults in the United States
Source: PLoS One. 2016 Jan 11;11(1):e0146119. doi: 10.1371/journal.pone.0146119 (PMC4708981; doi:10.1371/journal.pone.0146119)
Supplement: S1 File — (PDF) [file pone.0146119.s001.pdf]

Appendix

Individual-level Analyses

Appendix Table A. Adjusted risk ratios (RR) and 95% confidence intervals (95% CI) for the association between baseline CD4 count and baseline ART receipt with retention, by region, using region-by-baseline factor interactions.

Factor	RR	95% CI	P-value
Northeast-by-CD4+ Cell Count at Cohort Entry (cells/mm^3)[a]			
<200	Reference		
200-349	**1.03**	(1.00, 1.06)	0.02
350-499	1.03	(1.00, 1.06)	0.07
≥500	**1.03**	(1.00, 1.05)	0.02
Midwest-by-CD4+ Cell Count at Cohort Entry (cells/mm^3)[a]			
<200	Reference		
200-349	1.02	(0.98, 1.07)	0.33
350-499	1.01	(0.96, 1.06)	0.77
≥500	1.01	(0.98, 1.05)	0.45
South-by-CD4+ Cell Count at Cohort Entry (cells/mm^3)[a]			
<200	Reference		
200-349	**0.97**	(0.95, 0.99)	0.00
350-499	**0.96**	(0.94, 0.99)	0.00
≥500	**0.96**	(0.94, 0.98)	0.00
West-by-CD4+ Cell Count at Cohort Entry (cells/mm^3)[a]			
<200	Reference		
200-349	**0.96**	(0.94, 0.99)	0.00
350-499	**0.96**	(0.94, 0.99)	0.01
≥500	**0.94**	(0.92, 0.97)	0.00
Northeast-by-ART Receipt (≥1 month/year) at cohort entry			
No	Reference		
Yes	**1.22**	(1.17, 1.27)	0.00
Midwest-by-ART Receipt (≥1 month/year) at cohort entry			
No	Reference		
Yes	**1.30**	(1.21, 1.39)	0.00
South-by-ART Receipt (≥1 month/year) at cohort entry			
No	Reference		
Yes	**1.30**	(1.26, 1.34)	0.00
West-by-ART Receipt (≥1 month/year) at cohort entry			
No	Reference		
Yes	**1.24**	(1.19, 1.30)	0.00

All estimates are from fully adjusted models including age, sex, race, HIV risk, and time in care.

Appendix Figure A. a,b,c,d,e,f. Violin plots showing the smoothed kernel density of the distribution of predicted probabilities of retention over box-plots for the same distribution, at the individual-level by **(a.)** age category, **(b.)** sex, **(c.)** race/ethnicity, **(d.)** risk, and box-plots by **(e.)** ZCTA, **(f.)** state, and region of residence.

a.

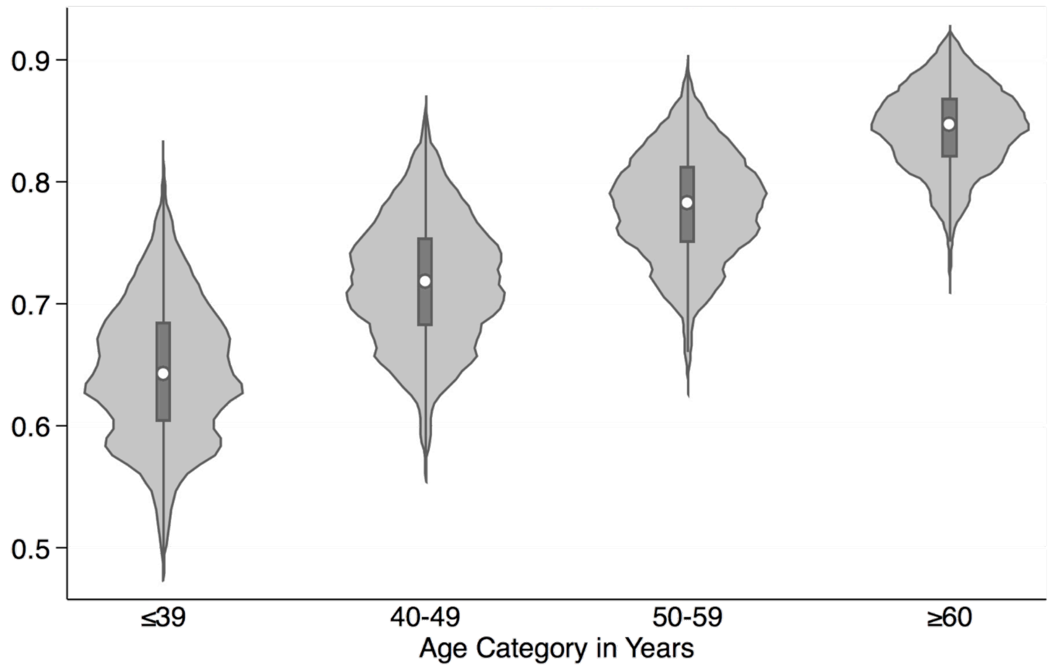

b.

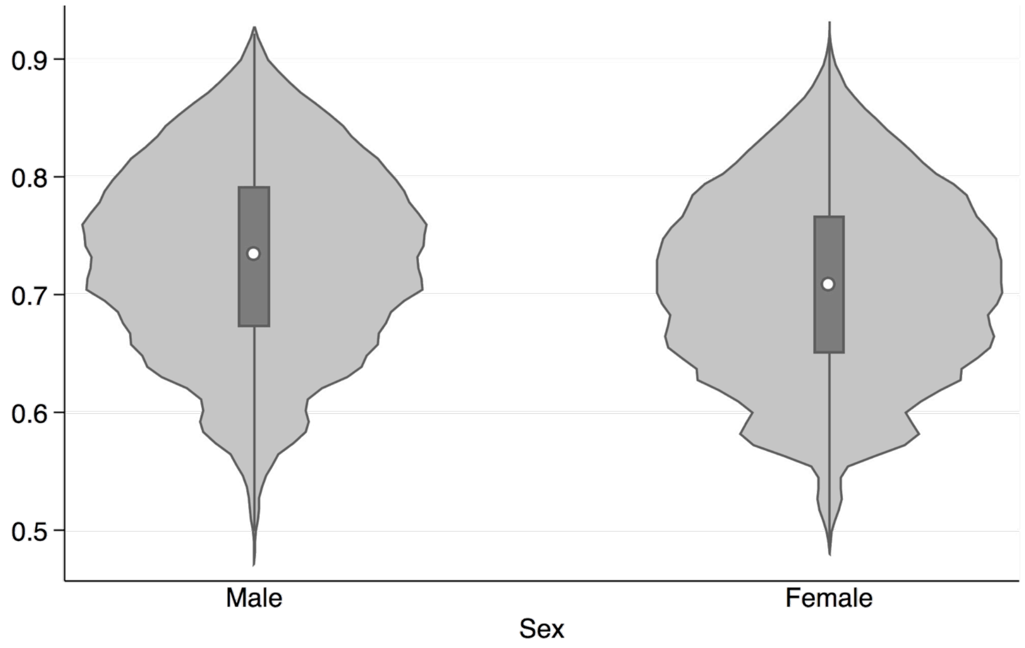

c.

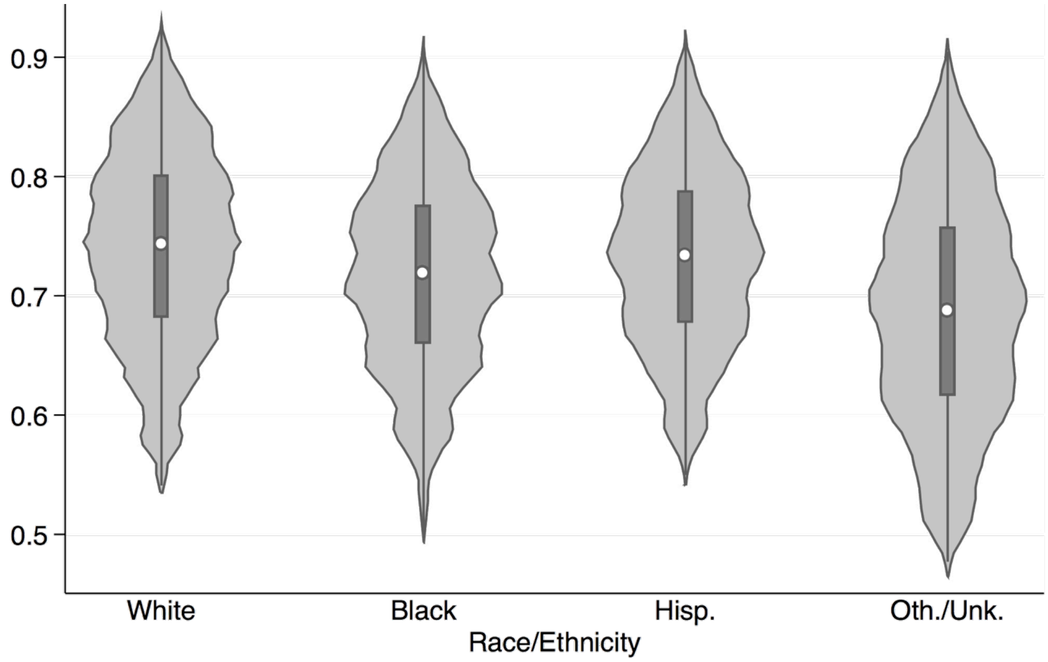
0.9
0.8
0.7
0.6
0.5
White
Black
Hisp.
Oth./Unk.
Race/Ethnicity

d.

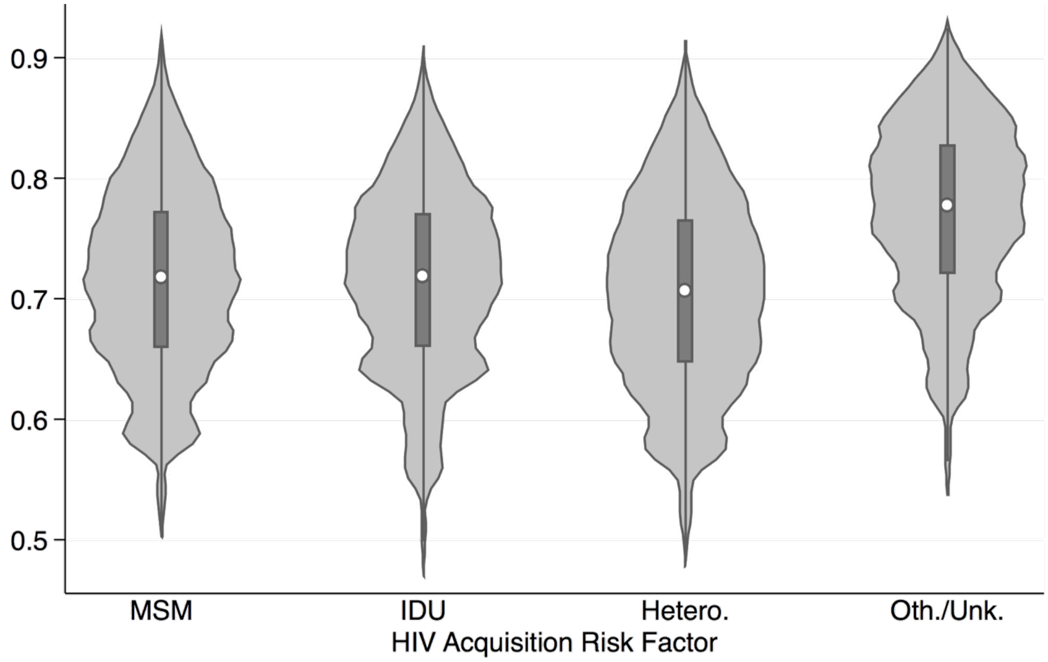
0.9
0.8
0.7
0.6
0.5
MSM
IDU
Hetero.
Oth./Unk.
HIV Acquisition Risk Factor

e.

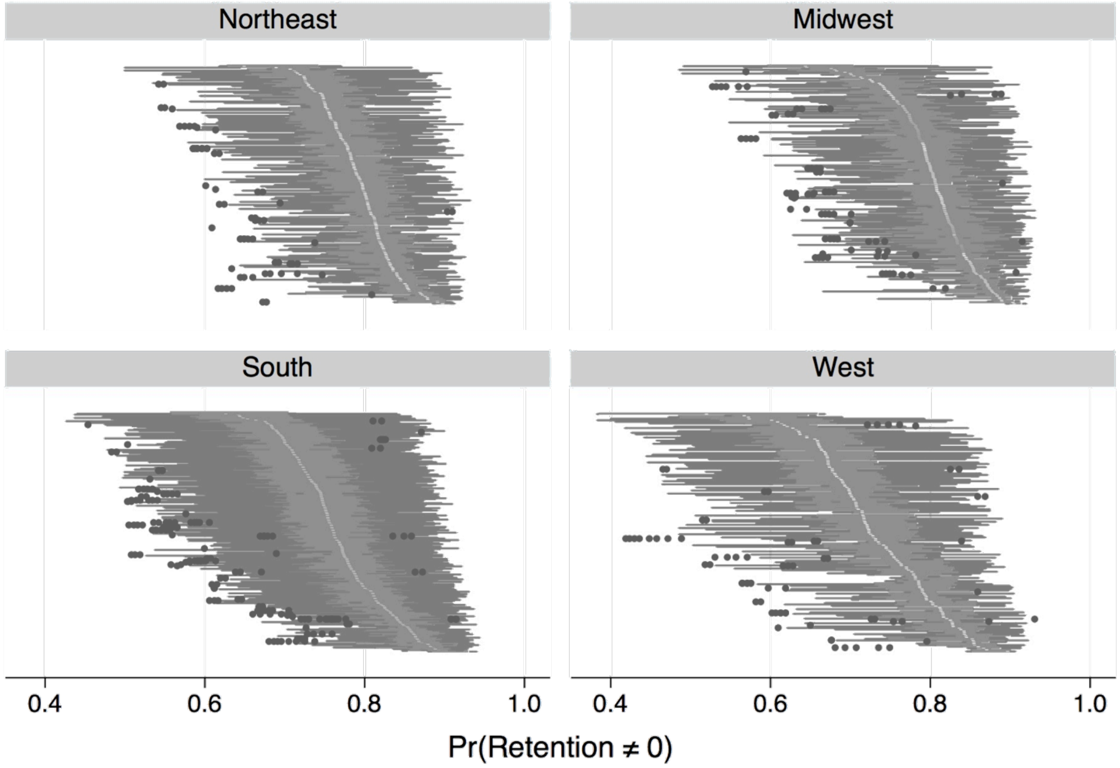
Northeast
Midwest
South
West
0.4
0.6
0.8
1.0
0.4
0.6
0.8
1.0
Pr(Retention ≠ 0)

f.

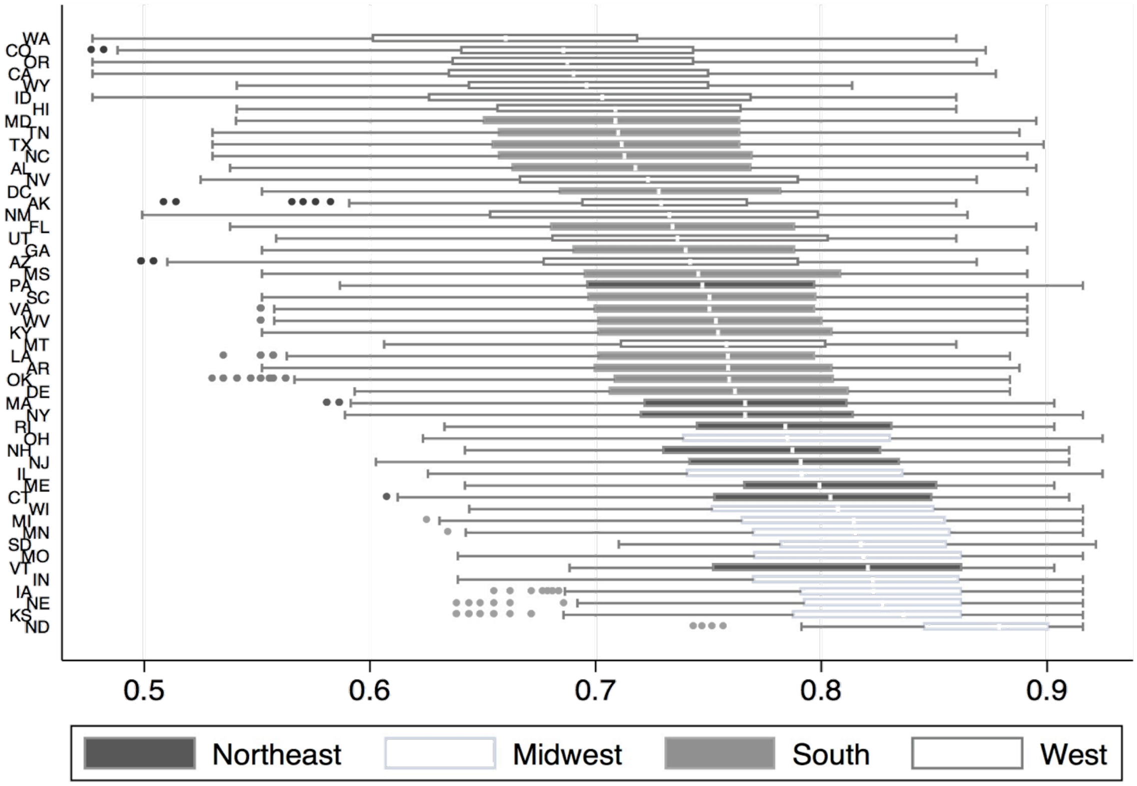
WA
CO
OR
CA
WY
ID
HI
MD
TN
TX
NC
AL
NV
DC
AK
NM
FL
UT
GA
AZ
MS
PA
SC
VA
WV
KY
MT
LA
AR
OK
DE
MA
NY
RI
OH
NH
NJ
IL
ME
CT
WI
MI
MN
SD
MO
VT
IN
IA
NE
KS
ND
0.5
0.6
0.7
0.8
0.9
Northeast
Midwest
South
West

Predicted probabilities are from a fully adjusted modified Poisson regression model with a Generalized Estimating Equation.

White circles inside violin plots indicate medians.

ZCTA-level Analyses

Appendix Figure B. a,b,c. Comparison of observed with predicted retention probabilities from ZCTA-level logistic regression model fit using GEE and adjusting for total person-time accrued within the ZCTA, sample-aggregated median age, proportion with female sex, proportion of Black race, and proportion with IDU HIV risk factor, and census-derived median age, proportion with female sex, proportion of Black race, proportion that is a rural area, and proportion living below the Federal poverty line. Plots are (**a.**) Quantile-Quantile, (**b.**) Normal-Quantile, and (**c.**) Normal-Probability plots. Structural zeros (ZCTAs with no observed patients contributing data) are excluded.

a.

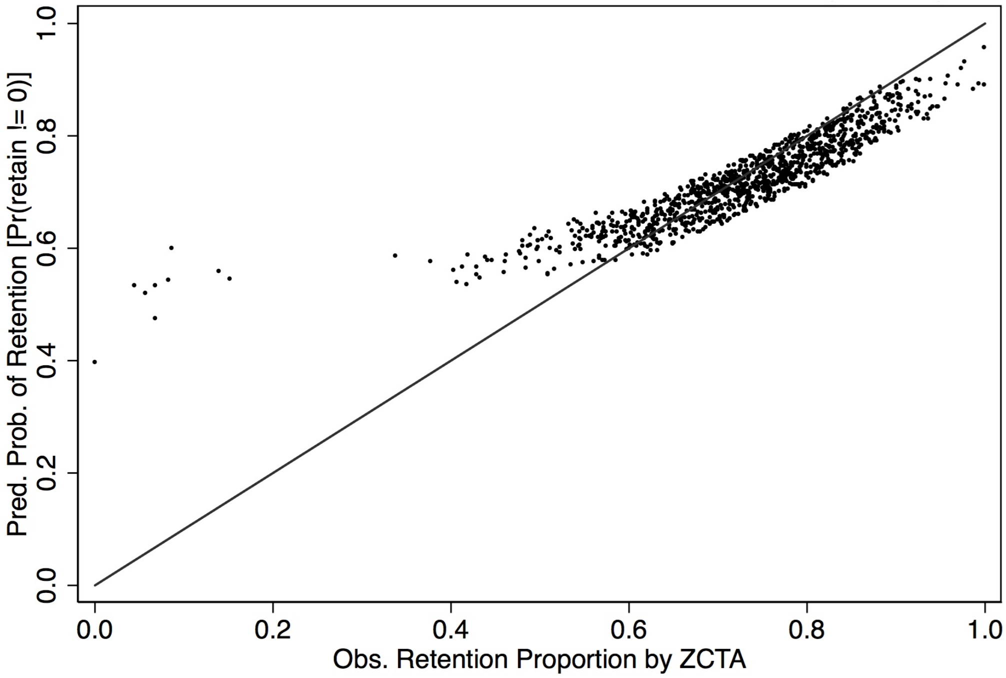

b.

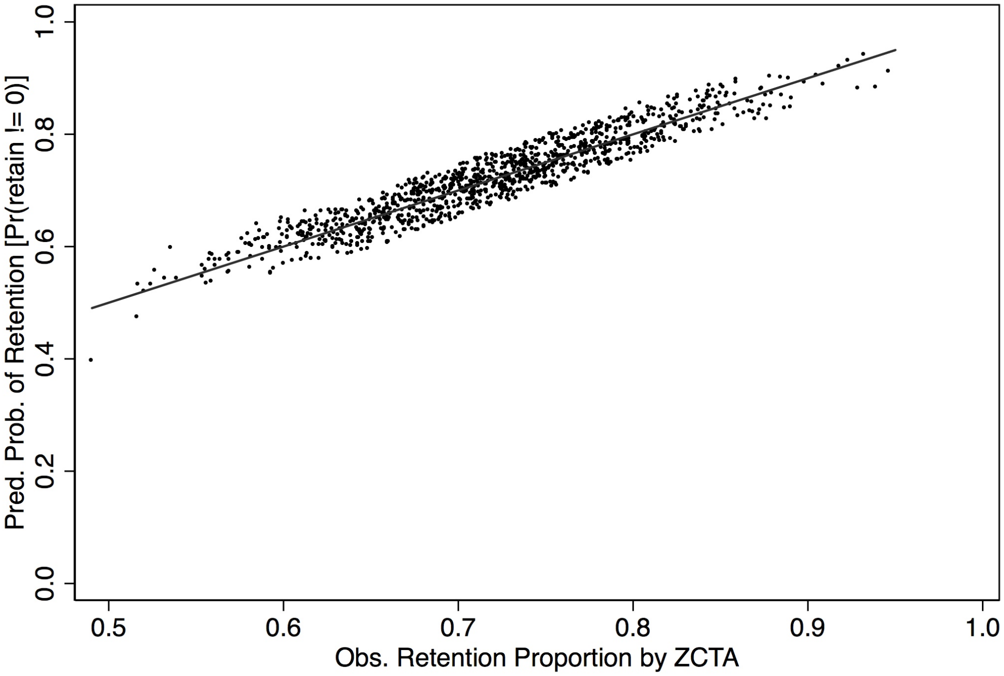
Pred. Prob. of Retention [Pr(retain != 0)]
0.0
0.2
0.4
0.6
0.8
1.0
0.5
0.6
0.7
0.8
0.9
1.0
Obs. Retention Proportion by ZCTA

c.

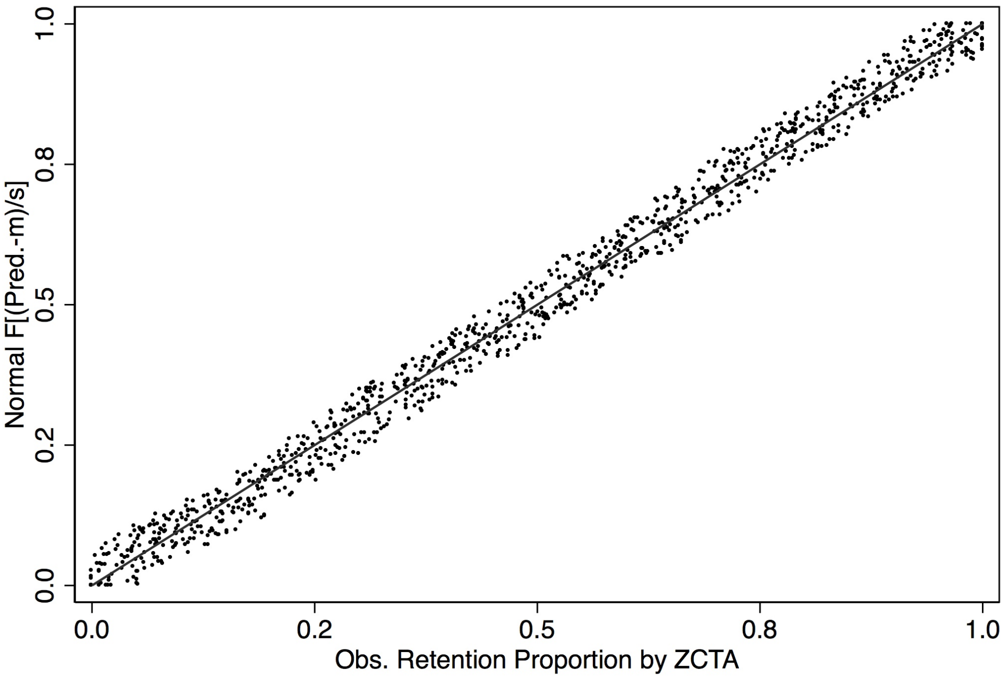
Normal F[(Pred.-m)/s]
0.0
0.2
0.5
0.8
1.0
0.0
0.2
0.5
0.8
1.0
Obs. Retention Proportion by ZCTA

Appendix Figure C. a,b,c,d,e. At the ZCTA-level, predicted linear fits are plotted by (a.) sample median age, (b.) census proportion with female sex, (c.) census proportion with Black race, (d.) census proportion of rural areas, and (e.) census proportion living below the Federal poverty line. Predicted probabilities are from logistic regression models with GEE.

a.

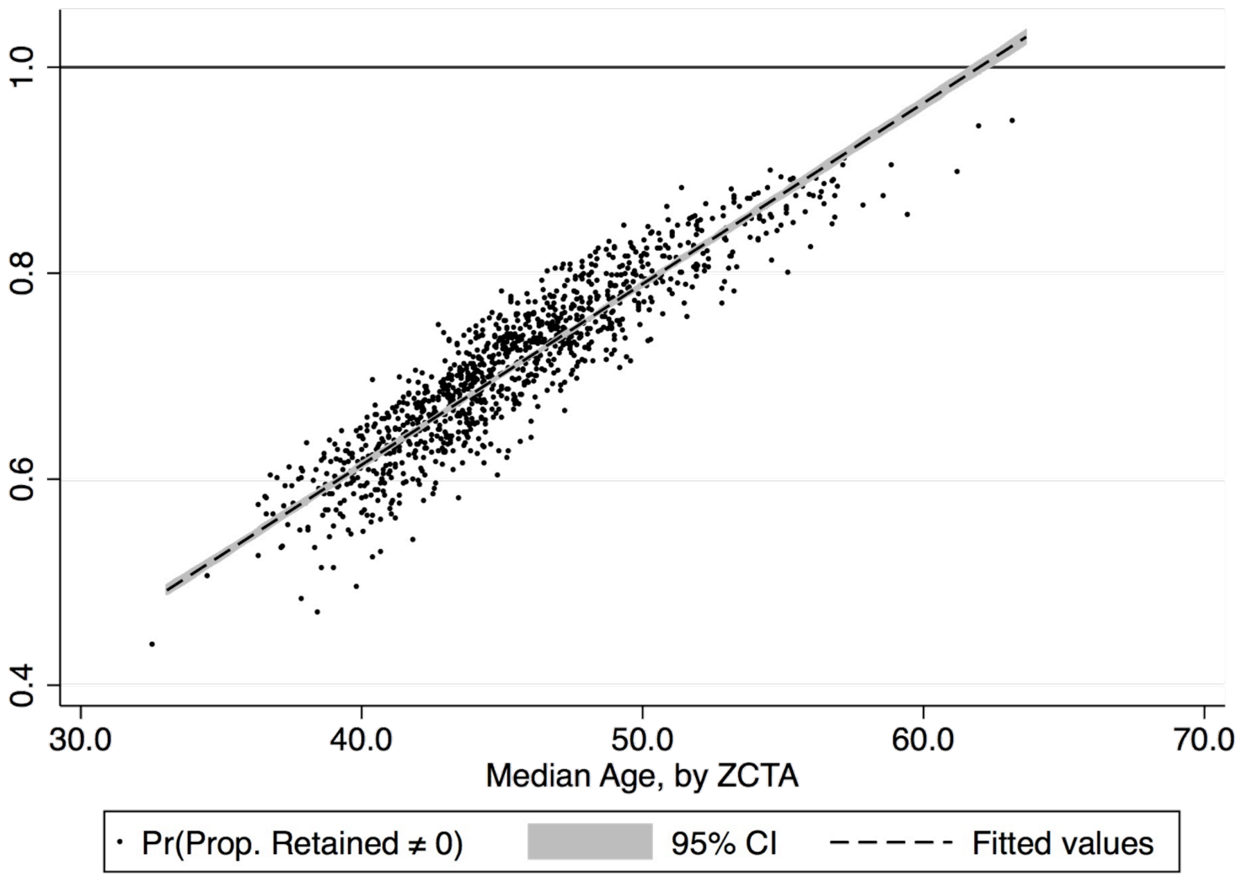

b.

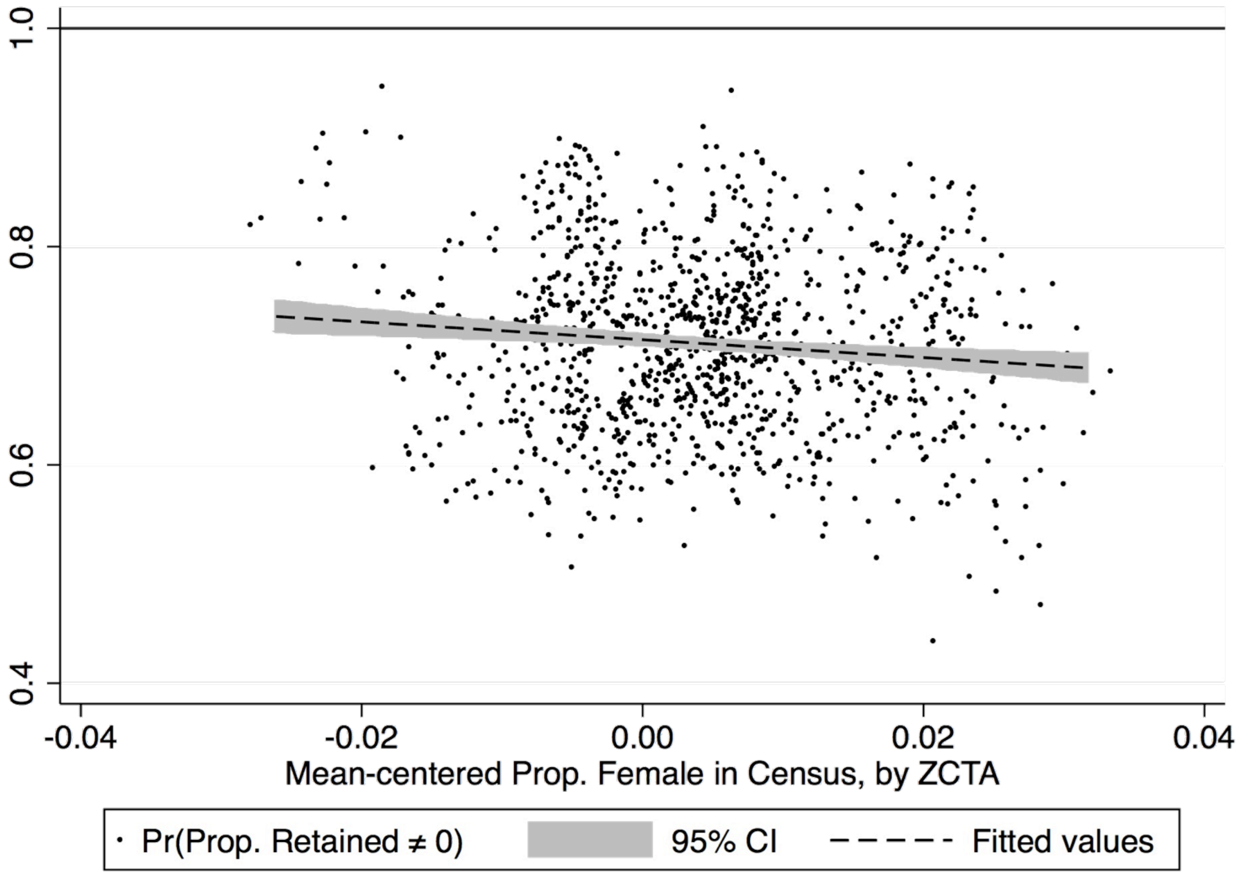
1.0
0.8
0.6
0.4
-0.04
-0.02
0.00
0.02
0.04
Mean-centered Prop. Female in Census, by ZCTA
Pr(Prop. Retained ≠ 0)
95% CI
Fitted values

c.

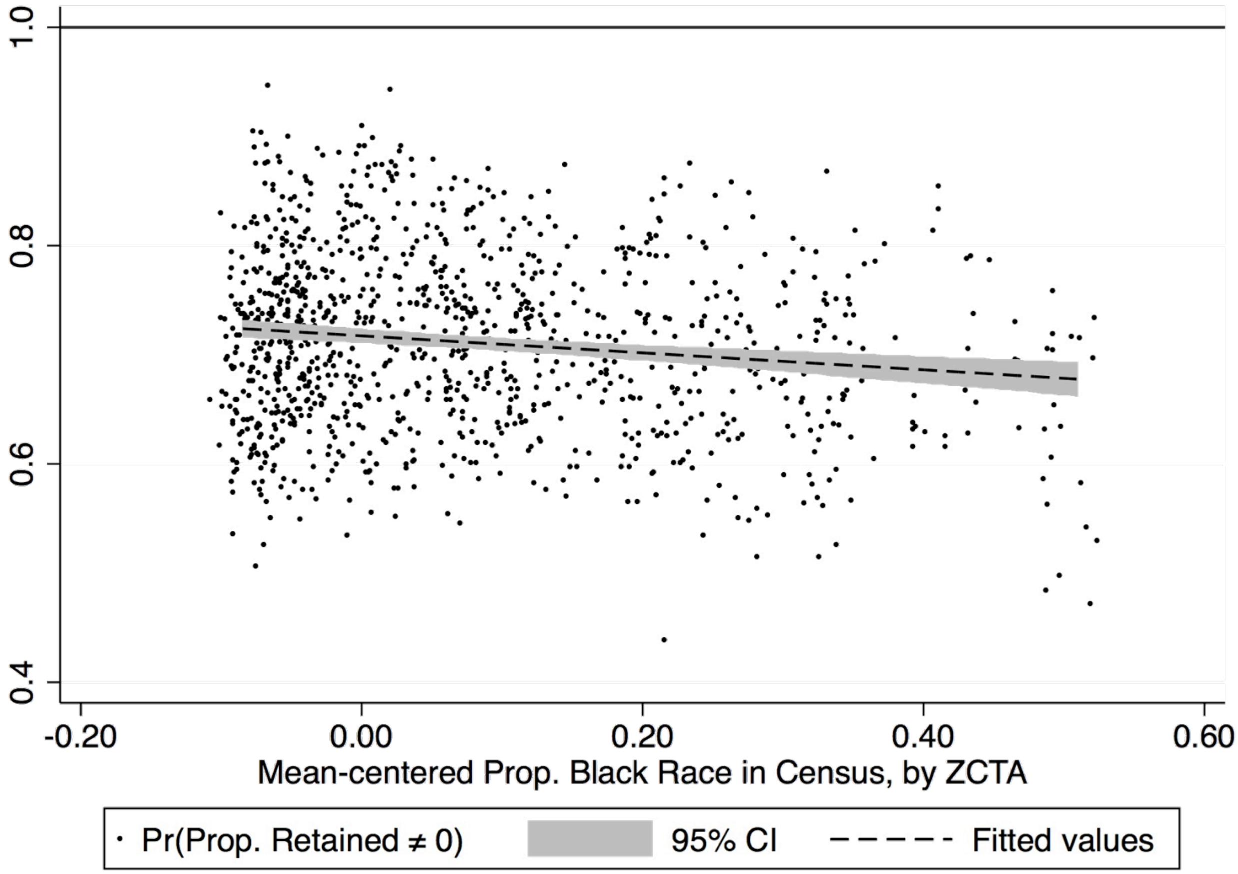
1.0
0.8
0.6
0.4
-0.20
0.00
0.20
0.40
0.60
Mean-centered Prop. Black Race in Census, by ZCTA
Pr(Prop. Retained ≠ 0)
95% CI
Fitted values

d.

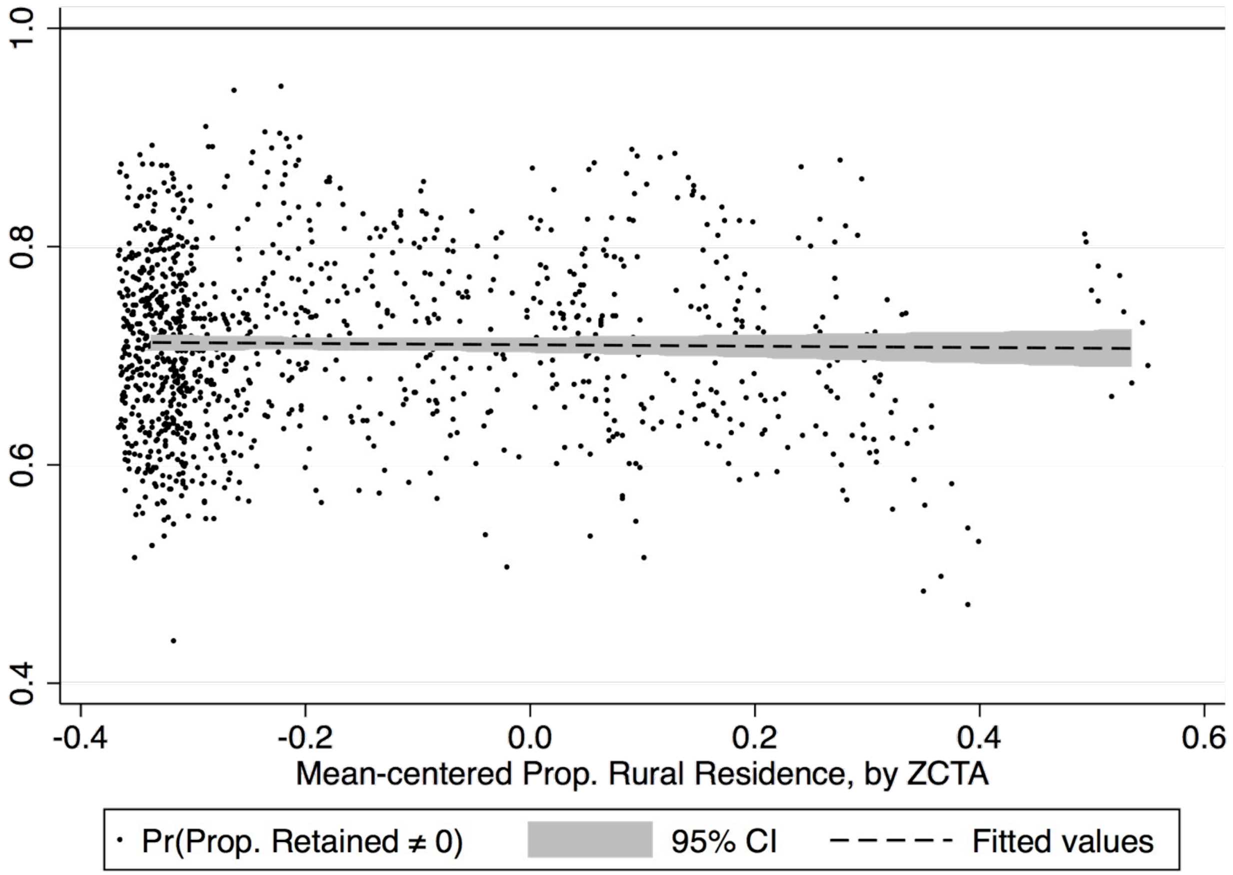
1.0
0.8
0.6
0.4
-0.4
-0.2
0.0
0.2
0.4
0.6
Mean-centered Prop. Rural Residence, by ZCTA
Pr(Prop. Retained ≠ 0)
95% CI
Fitted values

e.

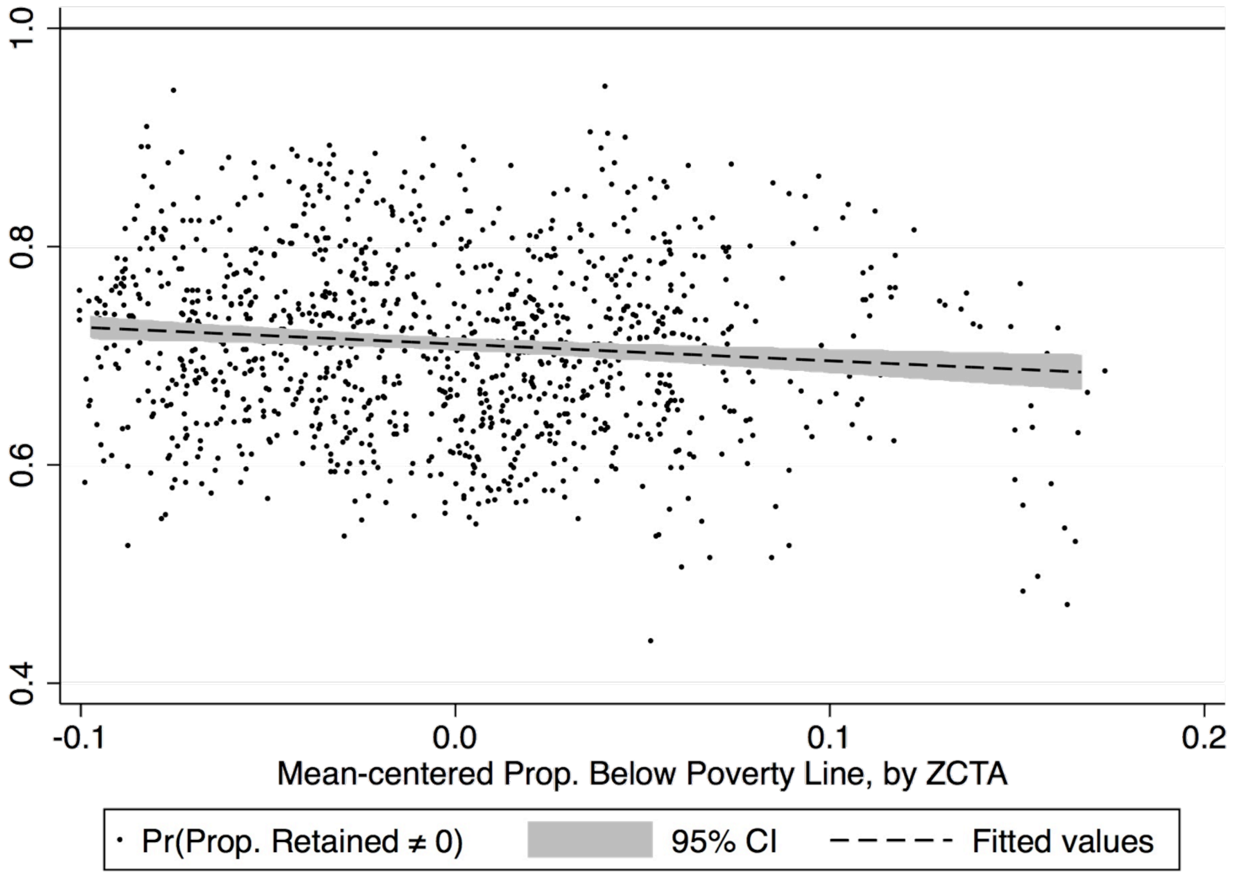
1.0
0.8
0.6
0.4
-0.1
0.0
0.1
0.2
Mean-centered Prop. Below Poverty Line, by ZCTA
Pr(Prop. Retained ≠ 0)
95% CI
Fitted values
